# Efficacy of terpenoids in attenuating pulmonary edema in acute lung injury: A meta-analysis of animal studies

**DOI:** 10.3389/fphar.2022.946554

**Published:** 2022-08-10

**Authors:** Shuai Wang, Sean X. Luo, Jing Jie, Dan Li, Han Liu, Lei Song

**Affiliations:** ^1^ Department of Vascular Surgery, General Surgery Center, The First Hospital of Jilin University, Chasngchun, JL, China; ^2^ Center for Pathogen Biology and Infectious Diseases, Key Laboratory of Organ Regeneration and Transplantation of the Ministry of Education, Department of Respiratory Medicine, State Key Laboratory for Zoonotic Diseases, The First Hospital of Jilin University, Changchun, China

**Keywords:** acute lung injury, terpenoids, lipopolysaccharide, lung wet-to-dry weight ratio, animal model

## Abstract

**Background:** The clinical efficiency of terpenoids in treating human acute lung injury (ALI) is yet to be determined. The lipopolysaccharide-induced rat model of ALI is a well-established and widely used experimental model for studying terpenoids’ effects on ALI. Using a systematic review and meta-analysis, the therapeutic efficiency of terpenoid administration on the lung wet-to-dry weight ratio in rats was investigated.

**Methods:** Using the Cochrane Library, Embase, and PubMed databases, a comprehensive literature search for studies evaluating the therapeutic efficacy of terpenoids on ALI in rats was conducted. The lung wet-to-dry weight ratio was extracted as the main outcome. The quality of the included studies was assessed using the Systematic Review Center for Laboratory Animal Experimentation’s risk of bias tool.

**Results:** In total, 16 studies were included in this meta-analysis. In general, terpenoids significantly lowered the lung wet-to-dry weight ratio when compared with the control vehicle (*p* = 0.0002; standardized mean difference (SMD): −0.16; 95% confidence interval (CI): −0.24, −0.08). Subgroup analysis revealed that low dose (≤10 μmol/kg) (*p <* 0.0001; SMD: −0.68; 95% CI: −1.02, −0.34), intraperitoneal injection (*p* = 0.0002; SMD: −0.43; 95% CI: −0.66, −0.20), diterpenoid (*p* = 0.004; SMD: −0.13; 95% CI: −0.23, −0.04), and triterpenoid (*p* = 0.04; SMD: −0.28; 95% CI: −0.54, −0.01) significantly lowered the lung wet-to-dry weight ratio when compared with the control vehicle.

**Conclusion:** A low dose of diterpenoid and triterpenoid administered intraperitoneally is effective in alleviating ALI. This systematic review and meta-analysis provides a valuable mirror for clinical research aiming at the advancement of terpenoids for preventive and therapeutic use.

**Systematic Review Registration:** CRD42022326779

## Introduction

Acute lung injury (ALI) is an acute inflammatory disease that disrupts the lung’s endothelial and epithelial barriers (Manicone, 2009). It is associated with systemic inflammatory response syndrome and multiple organ dysfunction syndrome (Wu et al., 2021). ALI, which is characterized by pulmonary edema and severe hypoxia, has also been regarded as the leading cause of death in patients with sepsis, imposing an enormous health burden worldwide each year (Schingnitz et al., 2010).

Although the pathophysiology of ALI has been extensively studied, effective clinical treatments for ALI remain limited. Therefore, there is an urgent need for the development of additional medications to treat ALI. Several natural compounds, such as terpenoids, alkaloids, and flavonoids, have been used over the last few years to treat ALI (Ren et al., 2019; Zhang et al., 2019; Zhao et al., 2021). Terpenoids, also known as isoprenoids, are the most abundant and structurally diverse natural compounds found in numerous plant species. They are a diverse and large group of naturally occurring organic chemicals derived from the 5-carbon compound isoprene. In addition, they are known to have diverse pharmacological properties, including antiatherosclerotic, antitumor, anti-inflammatory, antinociceptive, and antimalarial activities (Liu et al., 2019; Yuan et al., 2020; El-Baba et al., 2021; Sankhuan et al., 2022). The majority of research on terpenoids’ anti-ALI effects has focused on diterpenoids; however, there is no consensus on other terpenoids (Yang et al., 2011; Yang et al., 2014; Li et al., 2018a).

Endotoxin can enter humans’ airways through inhalation of contaminated dust or aerosol particles in hospital, occupational, agricultural, and domestic environments. Lipopolysaccharide (LPS), which is a major endotoxin component of Gram-negative bacteria, is regarded as the most important pathogen responsible for the development of ALI in sepsis (Li et al., 2014a; Park et al., 2018). Animal studies allow for the investigation of the efficacy and safety of novel therapies, linking basic research and clinical trials. LPS is thought to be a significant inducer of lung injury. Because of its widespread use and accessibility, LPS-induced lung injury is the most commonly used animal model of ALI for replicating the pathophysiological process (Li et al., 2018a). In this present meta-analysis, we investigated the effects of terpenoid administration on the wet-to-dry weight (W/D) ratio of the lungs in rats with LPS-induced ALI to better understand the preventive and therapeutic potential of terpenoids on ALI.

## Materials and methods

### Reporting standards

The systematic review protocol for animal intervention studies was prepared in accordance with the Preferred Reporting Items for Systematic Reviews and Meta-Analyses guideline and the Systematic Review Center for Laboratory Animal Experimentation (SYRCLE) format (Hooijmans and Ritskes-Hoitinga, 2013; De Vries et al., 2015).

### Search strategy

A comprehensive search was conducted by a competent information specialist (SW) in the Cochrane Library, Embase, and PubMed databases between January 2000 and March 2022 using the terms “acute lung injury,” “ALI,” “terpenoid,” “lipopolysaccharide,” “LPS,” and “rat.” The search terms are as follows: (acute lung injury or ALI) and (diterpenoid or hemiterpenoid or monoterpenoid or polyterpenoid or sesquiterpenoid or sesterterpenoid or terpenoid or tetraterpenoid or triterpenoid) and (lipopolysaccharide or LPS) and (rat or rats). Following a manual screening, further relevant studies were identified from the datasheet of included and reviewed articles.

### Inclusion and exclusion criteria

The inclusion criteria are as follows: (a) original research, (b) terpenoid intervention, and (c) rat with LPS-induced ALI research model. The exclusion criteria were as follows: (a) reviews, abstracts, case reports, comments, and editorials; (b) missing data; (c) duplicate and/or overlapping datasets; and (d) publications that were not written in English.

### Study selection

To collect qualified studies, the abstracts and titles of the articles identified by the comprehensive search were independently reviewed by three investigators (SW, JJ, and HL). The full text of potentially eligible studies was thereafter reviewed and checked by three investigators (SL, DL, and LS) to determine if the studies met the inclusion and exclusion criteria. Disagreements as regards the study’s selection were resolved through discussion and compromise.

### Data extraction

The characteristic data were extracted from qualified studies independently by three investigators (SL, LS, and HL), such as publication year, first author name, sample size of control and terpenoid groups, age, gender, rat strain and weight, diet type, terpenoid dosage, the interval between LPS administration and sacrifice, route and duration of LPS and terpenoid administration, lung wet-to-dry weight ratio, and dryer parameter. The statistics displayed graphically in the original publications were extracted using Adobe Photoshop (Ps v7.0). The main outcome was the lung wet-to-dry weight ratio, which is measured as a numerical value. Disagreements as regards data extraction were resolved through discussion and compromise.

### Quality assessment

The quality of the included studies was assessed by two investigators (SW and JJ) using SYRCLE’s risk of bias tool. The allocation concealment, incomplete outcome data, randomization, blinding and selective outcome assessments, baseline characteristics, random housing, domains evaluating sequence generation, and other sources of bias were included in the SYRCLE’s risk of bias tool (Hooijmans et al., 2014). Publication bias was assessed through visual inspection of funnel plots. Disagreements about quality assessment were resolved through discussion and compromise.

### Data synthesis and statistical analysis

All statistical analyses were conducted using the Review Manager (RevMan v5.3) software. The effects of control vehicle and terpenoids on lung wet-to-dry weight ratio were assessed using the mean differences with 95% confidence intervals (CI). A fixed effects model was used to pool studies, and the inconsistency index was used to calculate heterogeneity as high (*I* ^2^ ≥ 50%) or moderate (*I* ^2^ ≥ 30%).

In one study, datasets with more than three independent groups, namely, a control group, a low terpenoid dosage group, and a high terpenoid dosage group, were defined. A single control group was compared with various groups that investigated different terpenoid dosages in eight studies (Yang et al., 2011; Chen et al., 2014; Yang et al., 2014; Wei and Wang, 2017; Li et al., 2018a; Ren et al., 2019; Wang et al., 2021; Wu et al., 2021). To avoid a redundant expansion in the meta-analysis sample size, the number of samples in the control group in each study was divided by those in the matched groups. Subanalyses of the effects of different terpenoid routes and doses on lung wet-to-dry weight ratio were conducted. A sensitivity analysis was conducted to assess the robustness of the results. A *p-*value of <0.05 was considered statistically significant.

## Results

### Study selection

**FIGURE 1:**
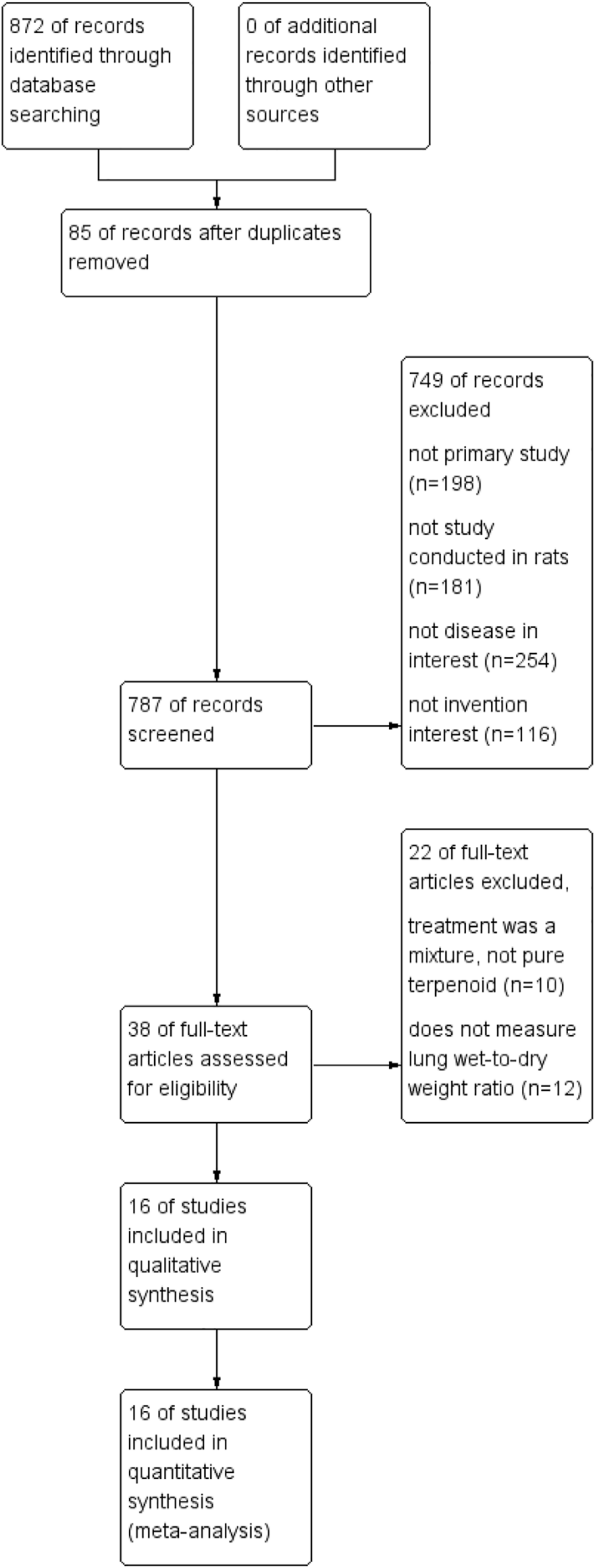
The flow diagram of the study identification and selection process.

Through the search strategy, 872 articles were identified. The investigators extracted the abstracts and titles and identified 38 studies that met the inclusion criteria. After reviewing all of the publications, 10 studies were excluded because of missing outcome data (Ehrhart et al., 2000; Tawadros et al., 2007; Nader and Baraka, 2012; An et al., 2014; Wang et al., 2015; Liu and Chen, 2016; Shen et al., 2017; Ni et al., 2019; Wang et al., 2019; Yang et al., 2019), and another 12 studies were excluded because multiple interventions were investigated (Murakami et al., 2000; Lin et al., 2011; Li et al., 2014b; Li et al., 2016a; Li et al., 2016b; Baradaran Rahimi et al., 2019; Ye et al., 2019; Duan et al., 2020; Yue et al., 2020; Choi et al., 2021; Zhang et al., 2021; Liu et al., 2022). In conclusion, 16 studies were included in this meta-analysis, as described below (Shi et al., 2007; Yang et al., 2011; Chen et al., 2014; Yang et al., 2014; Yuan et al., 2014; Wei and Wang, 2017; Li et al., 2018a; Li et al., 2018b; Luo et al., 2019; Ren et al., 2019; Zhang et al., 2020; Ali et al., 2021; Dikmen et al., 2021; Li et al., 2021; Wang et al., 2021; Wu et al., 2021) (Figure 1).

### Study characteristics

From the 16 included studies, 29 datasets and 499 rats were extracted. The characteristics of these studies are shown in Table 1. Rats ranged in age from 6 to 8 weeks and in weight from 90 to 350 g. In terms of intervention, diterpenoid, monoterpenoid, sesquiterpenoid, and triterpenoid were used in seven studies (Shi et al., 2007; Yang et al., 2011; Yang et al., 2014; Wei and Wang, 2017; Li et al., 2018a; Li et al., 2018b; Luo et al., 2019), three studies (Yuan et al., 2014; Ren et al., 2019; Li et al., 2021), two studies (Ali et al., 2021; Wang et al., 2021), and four studies (Chen et al., 2014; Zhang et al., 2020; Dikmen et al., 2021; Wu et al., 2021), respectively. In addition, 13 studies used male rats (Shi et al., 2007; Yang et al., 2011; Chen et al., 2014; Yang et al., 2014; Yuan et al., 2014; Wei and Wang, 2017; Li et al., 2018a; Luo et al., 2019; Ren et al., 2019; Zhang et al., 2020; Dikmen et al., 2021; Li et al., 2021; Wang et al., 2021), whereas three studies did not report gender (Li et al., 2018b; Ali et al., 2021; Wu et al., 2021). Sprague Dawley rats were used in 11 studies (Shi et al., 2007; Yang et al., 2014; Wei and Wang, 2017; Li et al., 2018a; Li et al., 2018b; Luo et al., 2019; Ren et al., 2019; Ali et al., 2021; Li et al., 2021; Wang et al., 2021; Wu et al., 2021) and Wistar rats in five studies (Yang et al., 2011; Chen et al., 2014; Yuan et al., 2014; Zhang et al., 2020; Dikmen et al., 2021).

**TABLE 1:** The characteristics of included studies.

Study	Terpenoid	Gender	Age	Strain	Weight	Route of LPS	Dose of LPS	Dose of terpenoid	Interval between LPS administration and sacrifice	Route of terpenoid	Parameter of dryer	Groups and sample size
Ali F, 2021 (Ali et al., 2021)	Aescin, triterpenoid	Male	8 w	Wistar	180–200 g	i.t.	8 mg/kg	4.42 µmol/kg	24 h	i.g.	80°C	Control = 10
											24 h	Triterpenoid = 10
Chen J, 2014 (Chen et al., 2014)	Triptolid, diterpenoid	Male	?	Sprague Dawley	200–250 g	i.v.	5 mg/kg	0.28 µmol/kg	12 h	i.p.	?	Control = 5
								0.14 µmol/kg				Diterpenoid high = 5
								0.08 µmol/kg				Diterpenoid medium = 5
												Diterpenoid low = 5
Dikmen N, 2021 (Dikmen et al., 2021)	Oleuropein, monoterpenoid	Male	8–10 w	Wistar	180–250 g	i.t.	5 mg/kg	370 µmol/kg	20 h	i.g.	60°C	Control = 8
											72 h	Monoterpenoid = 8
Li J, 2017 (Li et al., 2018a)	Tanshinone IIA, diterpenoid	Male	10 w	Wistar	250–300 g	i.v.	5 mg/kg		24 h	i.v.		Control = 24
								34 µmol/kg			70 °C	Diterpenoid high = 24
								20 µmol/kg			72 h	Diterpenoid medium = 15
								10 µmol/kg				Diterpenoid low = 15
Li L, 2018 (Li et al., 2018b)	Tanshinone IIA, diterpenoid	Male	8 w	Sprague Dawley	200–220 g	i.p.	10 mg/kg	1 µmol/kg	8 d	i.p.	?	Control = 10
												Diterpenoid = 10
Li S, 2021 (Li et al., 2021)	Retinoic acid, diterpenoid	Male	8–10 w	Sprague Dawley	220–270 g	i.v.	5 mg/kg	17 µmol/kg	48 h	i.p.	70°C	Control = 10
											72 h	Diterpenoid = 10
Luo X, 2019 (Luo et al., 2019)	Genipin, monoterpenoid	Male	8 w	Sprague Dawley	180–220 g	i.t.	5 mg/kg	22 µmol/kg	12 h	i.t.	80°C	Control = 6
											48 h	Monoterpenoid = 6
Shi XM, 2007 (Shi et al., 2007)	Tanshinone IIA, diterpenoid	?	?	Sprague Dawley	240–280 g	i.v.	5 mg/kg	17 µmol/kg	6 h	i.p.	80°C	Control = 8
											20 h	Sesquiterpenoid = 8
Wang YJ, 2020 (Wang et al., 2021)	Zaluzanin D, sesquiterpenoid	Male	6–8 w	Sprague Dawley	?	i.t.	3 mg/kg		7 d	i.v.		Control = 6
								347 µmol/kg			60°C	Sesquiterpenoid high = 6
								174 µmol/kg			48 h	Sesquiterpenoid medium = 6
								69 µmol/kg				Sesquiterpenoid low = 6
Wei Y, 2017 (Wei and Wang, 2017)	Celastrol, triterpenoid	Male	?	Wistar	180–220 g	i.t.	2 mg/kg		24 h	i.g.		Control = 8
								44 µmol/kg			80°C	Triterpenoid high = 8
								11 µmol/kg			72 h	Triterpenoid medium = 8
								1.1 µmol/kg				Triterpenoid low = 8
Wu Y, 2021 (Wu et al., 2021)	Platycodin D, triterpenoid	?	6 w	Sprague Dawley	90–110 g	i.t.	5 mg/kg		?	i.p.		Control = 7
								20 µmol/kg			60°C	Triterpenoid high = 7
								10 µmol/kg			48 h	Triterpenoid low = 7
Yang N, 2014 (Yang et al., 2014)	Andrographolide, diterpenoid	Male	8 w	Sprague Dawley	180–220 g	i.v.	5 mg/kg		6 h	i.g.		Control = 43
								128 µmol/kg			80°C	Diterpenoid high = 43
								13 µmol/kg			72 h	Diterpenoid low = 43
Yang W, 2011 (Yang et al., 2011)	Isoforskolin, diterpenoid	Male	12 w	Sprague Dawley	260–300 g	i.v.	6 mg/kg		3 h	i.p.		Control = 8
								49 µmol/kg			80 °C	Diterpenoid high = 8
								24 µmol/kg			48 h	Diterpenoid medium = 8
								12 µmol/kg				Diterpenoid low = 8
Yuan Q, 2014 (Yuan et al., 2014)	Ginsenoside Rb1, triterpenoid	Male	?	Wistar	300–350 g	i.v.	0.1 mg/kg	4.5 µmol/kg	?	i.v.	80°C	Control = 10
											48 h	Triterpenoid = 10
Zhang E, 2020 (Zhang et al., 2020)	Artesunate, sesquiterpenoid	?	?	Sprague Dawley	220–250 g	i.t	5 mg/kg	5.2 µmol/kg	24 h	i.p.	?	Control = 8
												Sesquiterpenoid = 8
Zhang Z, 2019 (Zhang et al., 2019)	Genipin, monoterpenoid	Male	8 w	Sprague Dawley	180–220 g	i.t.	5 mg/kg		12 h	i.p.		Control = 6
								22 µmol/kg			80°C	Monoterpenoid high = 6
								8.8 µmol/kg			48 h	Monoterpenoid low = 6

Note: i.v., intravenous injection; i.t., intratracheal administration; i.p., intraperitoneal injection; i.g., intragastric administration;? = not reported.

LPS was administered intravenously in seven studies (Yang et al., 2011; Yang et al., 2014; Wei and Wang, 2017; Li et al., 2018a; Li et al., 2018b; Luo et al., 2019; Dikmen et al., 2021), intraperitoneally in one study (Shi et al., 2007), and intratracheally in eight studies (Chen et al., 2014; Yuan et al., 2014; Ren et al., 2019; Zhang et al., 2020; Ali et al., 2021; Li et al., 2021; Wang et al., 2021; Wu et al., 2021). The LPS dosage ranged from 0.1 to 10 mg/kg. Terpenoid was administered intragastrically in four studies (Chen et al., 2014; Yang et al., 2014; Yuan et al., 2014; Zhang et al., 2020), intravenously in three studies (Yang et al., 2011; Dikmen et al., 2021; Wang et al., 2021), intraperitoneally in eight studies (Shi et al., 2007; Wei and Wang, 2017; Li et al., 2018a; Li et al., 2018b; Luo et al., 2019; Ren et al., 2019; Ali et al., 2021; Wu et al., 2021), and intratracheally in one study (Li et al., 2021). The terpenoid dosage ranged from 0.08 to 370 μmol/kg. The terpenoid was given prior to LPS in eight studies (Chen et al., 2014; Yang et al., 2014; Yuan et al., 2014; Li et al., 2018a; Luo et al., 2019; Ren et al., 2019; Zhang et al., 2020; Li et al., 2021), after LPS in seven studies (Shi et al., 2007; Yang et al., 2011; Wei and Wang, 2017; Li et al., 2018b; Ali et al., 2021; Dikmen et al., 2021; Wang et al., 2021), and undetermined in one study (Wu et al., 2021). The interval between LPS administration and sacrifice ranged from 3 h to 8 days. The drying time ranged from 20 to 72 h, and the temperature ranged from 60 to 80°C.

### Quality assessment

**FIGURE 2:**
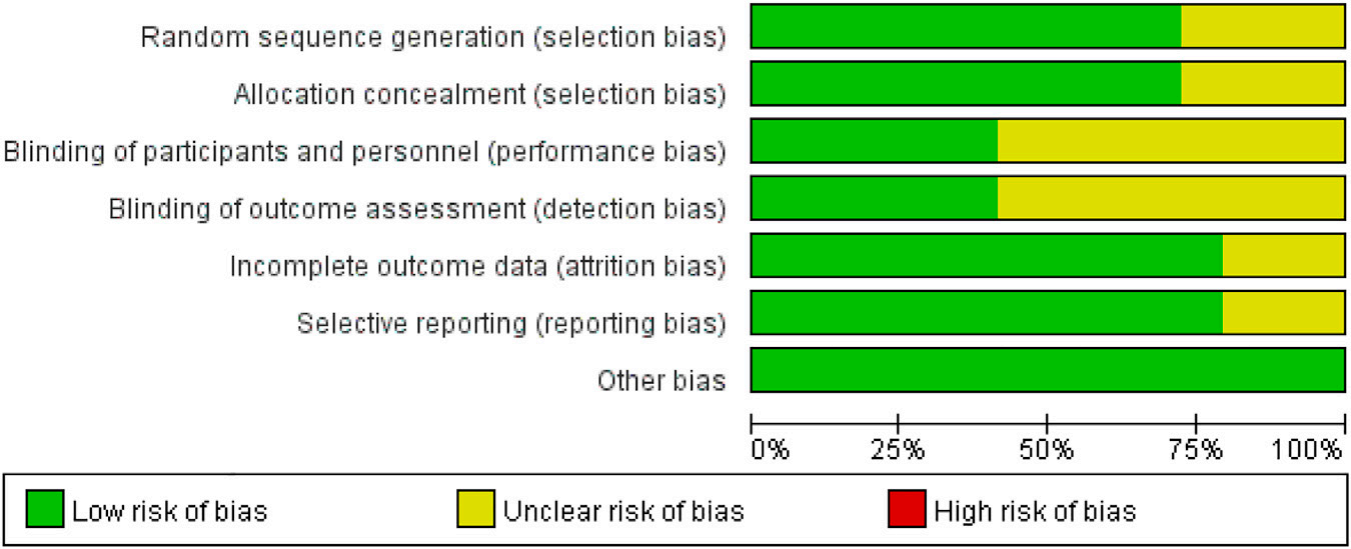
The risk of bias and quality evaluation score (%) per risk of bias item.

**FIGURE 3:**
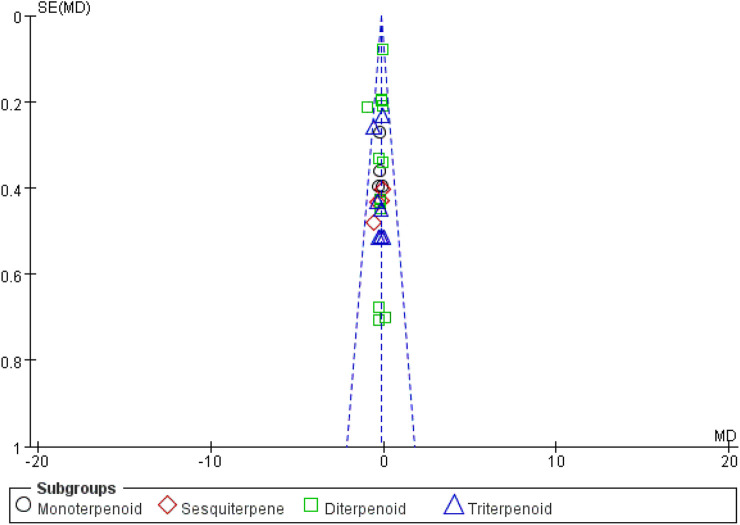
The funnel plot for accessing publication bias.

The quality assessment of these studies is shown in Figure 2. In total, 12 studies were randomized, with eight studies demonstrating unclear risks of bias in blinding and allocation concealment. All studies’ outcomes were reported, with six studies demonstrating an unclear risk of selective outcome reporting. Overall, the risk of bias from other sources was low. The potential publication bias was evaluated through visual inspection of a funnel plot (Figure 3).

### Effect of terpenoids on lung wet-to-dry weight ratio

**FIGURE 4:**
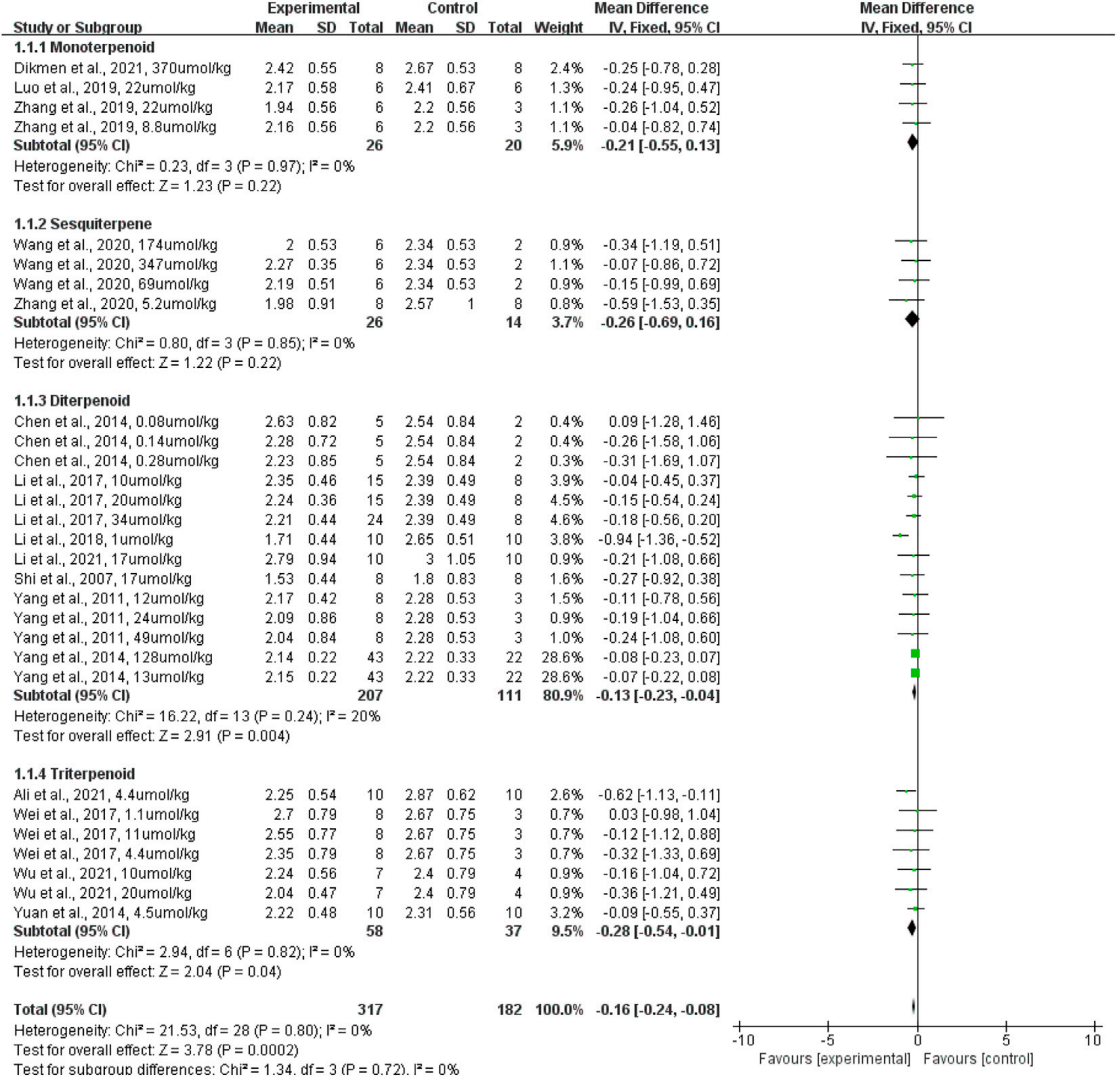
The forest plot of therapeutic efficiency of terpenoids on lung wet-to-dry weight ratio. Subgroup analyses investigated the therapeutic efficiency of monoterpenoid, sesquiterpene, diterpenoid, and triterpenoid. CI, confidence interval; IV, inverse variance; Std, standard; SD, standard deviation.

The effect of terpenoids on lung wet-to-dry weight ratio was presented for 29 datasets acquired from 16 studies (rats given terpenoids [*n* = 317] vs. rats given a control vehicle [*n* = 182]). Overall, terpenoids significantly reduced the lung wet-to-dry weight ratio when compared with the control vehicle (*p* = 0.0002; standardized mean difference (SMD): −0.16; 95% CI: −0.24, −0.08), with no evidence of heterogeneity among studies (*I* ^2^ = 0%) (Figure 4).

Subgroup analyses were conducted to assess the effects of diterpenoid, monoterpenoid, sesquiterpenoid, and triterpenoid on the lung wet-to-dry weight ratio. Monoterpenoid (rats given a monoterpenoid [*n* = 26] vs. rats given a control vehicle [*n* = 20]; *p* = 0.22; SMD: −0.21; 95% CI: −0.55, 0.13) and sesquiterpenoid (rats given a sesquiterpenoid [*n* = 26] vs. rats given a control vehicle [*n* = 14]; *p* = 0.22; SMD: −0.26; 95% CI: −0.69, 0.16) did not significantly lower the lung wet-to-dry weight ratio when compared with the control vehicle, with no evidence of heterogeneity among studies (*I* ^2^ = 0%). Diterpenoid (rats given a diterpenoid [*n* = 207] vs. rats given a control vehicle [*n* = 111]) significantly reduced the lung wet-to-dry weight ratio when compared with the control vehicle (*p* = 0.004; SMD: −0.13; 95% CI: −0.23, −0.04), with evidence of low heterogeneity among studies (*I* ^2^ = 20%). Triterpenoid (rats given a triterpenoid [*n* = 58] vs. rats given a control vehicle [*n* = 37]) significantly decreased the lung wet-to-dry weight ratio when compared with the control vehicle (*p* = 0.04; SMD: −0.28; 95% CI: −0.54, −0.01), with no evidence of heterogeneity among studies (*I* ^2^ = 0%) (Figure 4). The sensitivity analysis, which substituted the random effects model for the fixed effects model, had no effect on the overall outcome (SMD: −0.16, CI: −0.24, −0.08 vs. SMD: −0.36, CI: −0.55, −0.17).

**FIGURE 5:**
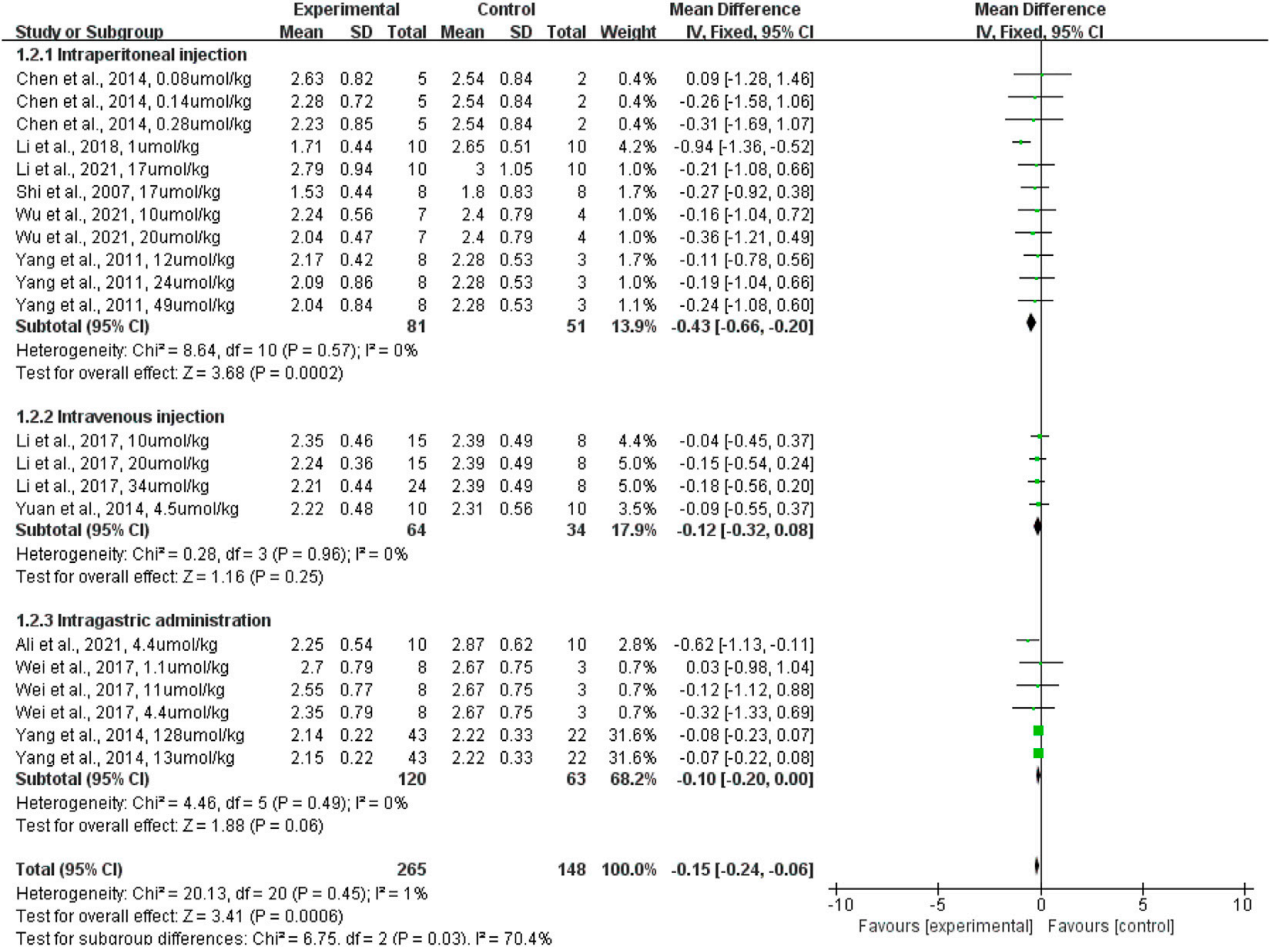
The forest plot of therapeutic efficiency of the route of terpenoid administration on lung wet-to-dry weight ratio. Subgroup analyses evaluated the therapeutic efficiency of intraperitoneal injection, intravenous injection, and intragastric administration. CI, confidence interval; IV, inverse variance; Std, standard; SD, standard deviation.

In terms of the administration route, subgroup analyses revealed that intraperitoneal injection of terpenoid (rats given a terpenoid [*n* = 81] vs. rats given a control vehicle [*n* = 51]) significantly reduced the lung wet-to-dry weight ratio when compared with the control vehicle (*p* = 0.0002; SMD: −0.43; 95% CI: −0.66, −0.20), with no evidence of heterogeneity among studies (*I* ^2^ = 0%). However, intravenous injection of terpenoid (rats given a terpenoid [*n* = 64] vs. rats given a control vehicle [*n* = 34]) did not significantly lower the lung wet-to-dry weight ratio when compared with the control vehicle (*p* = 0.25; SMD: −0.12; 95% CI: −0.32, −0.08), with no evidence of heterogeneity among studies (*I* ^2^ = 0%). Likewise, intragastric administration of terpenoid (rats given a terpenoid [*n* = 96] vs. rats given a control vehicle [*n* = 54]) did not significantly lower the lung wet-to-dry weight ratio when compared with the control vehicle (*p* = 0.06; SMD: −0.10; 95% CI: −0.20, −0.00), with no evidence of heterogeneity among studies (*I* ^2^ = 0%) (Figure 5). The sensitivity analysis, which substituted the random effects model for the fixed effects model, had no effect on the overall outcome (SMD: −0.15, CI: −0.24, −0.06 vs. SMD: −0.36, CI: −0.57, −0.15).

**FIGURE 6:**
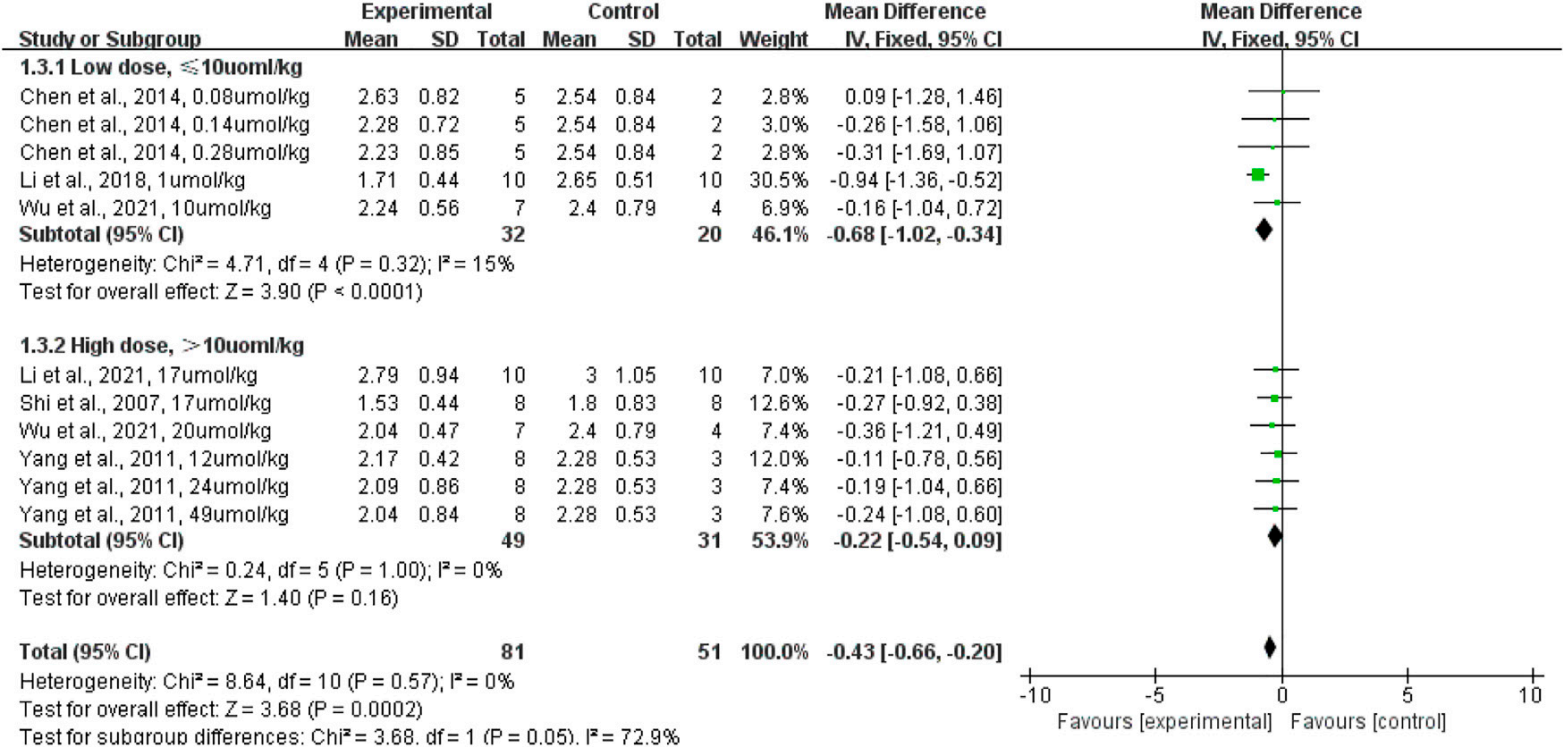
The forest plot of therapeutic efficiency of terpenoid dose on lung wet-to-dry weight ratio. Subgroup analyses investigated therapeutic efficiency of high dose (>10 µmol/kg) and low dose (≤10 µmol/kg). CI, confidence interval; IV, inverse variance; Std, standard; SD, standard deviation.

In terms of terpenoid dosage, subgroup analyses revealed that low doses of terpenoid administered intraperitoneally (rats given terpenoid at a dose of 10 μmol/kg or lower [*n* = 32] vs. rats given a control vehicle [*n* = 20]) significantly lowered the lung wet-to-dry weight ratio when compared with the control vehicle (*p <* 0.0001; SMD: −0.68; 95% CI: −1.02, −0.34), with no evidence of heterogeneity among studies (*I* ^2^ = 0%). However, the high dose (rats given terpenoid at a dose greater than 10 μmol/kg [*n* = 49] vs. rats given a control vehicle [*n* = 31]) did not significantly lower the lung wet-to-dry weight ratio when compared with the control vehicle (*p* = 0.16; SMD: −0.22; 95% CI: −0.54, −0.09), with no evidence of heterogeneity among studies (*I* ^2^ = 0%) (Figure 6). The sensitivity analysis, which substituted the random effects model for the fixed effects model, had no effect on the overall outcome (SMD: −0.43, CI: −0.66, −0.20 vs. SMD: −0.46, CI: −0.83, −0.09).

## Discussion

Despite significant advances in pharmacotherapy agents for ALI, such as antibiotics, N-acetylcysteine, β-agonists, corticosteroids, surfactants, and statins, an efficient approach to lowering ALI morbidity and mortality is yet to be identified (Lewis et al., 2019). Because diterpenoids were found to be promising in treating ALI in some studies (Yang et al., 2011; Yang et al., 2014; Li et al., 2018a), it was necessary to investigate the pharmacological effects of terpenoids on ALI. The use of animal models provides a valuable gateway for preclinical research, identifying novel therapeutic strategies for disease and developing new drugs. An ideal animal model should be able to replicate the consequences and mechanisms of human disease, including pathological and physiological hallmarks. LPS is a component of Gram-negative bacterial cell walls. LPS-induced animal models, by inhalation or systemic (intravenous and intraperitoneal) administration, reproduce acute damage to the lung epithelial and endothelial barriers, as well as acute inflammatory responses, in a short period of time (typically <48 h) (Matute-Bello et al., 2008). LPS-induced injury is a valuable *in vivo* experimental model that is similar to ALI and acute respiratory distress syndrome in humans. LPS is easy to use, and its outcomes are often replicable in experiments. Although mouse models of human disease are widely used because of the availability of specific reagents and the development of transgenic mice that can be administered to assess the physiological function of specific genes, animal size remains an important consideration when choosing an animal model for ALI. Because there is no difference between rats and mice in ALI animal models (Matute-Bello et al., 2008), rats were used as experimental animals in this study.

Previous animal experiments suggest that natural terpenoids may have therapeutic effects on ALI. However, study parameters such as sample size, animal strain and age, and treatment and follow-up duration were noted to differ among studies. A quantitative and comprehensive analysis of these heterogeneous sources of animal model data can provide insights into the benefits of terpenoids in the treatment of ALI. Therefore, we conducted a systematic review and meta-analysis to investigate the effects of terpenoids on lung wet-to-dry weight ratio in rats with LPS-induced ALI. The findings revealed that the use of terpenoids significantly reduced the lung wet-to-dry weight ratio and alleviated pulmonary edema when compared with the control group.

The terpenoid family includes monoterpenoid, sesquiterpenoid, diterpenoid, triterpenoid, and tetraterpenoid (Christianson, 2017). In a subgroup analysis, the number of isoprene groups, monoterpenoid and sesquiterpenoid, did not drastically lower the lung wet-to-dry weight ratio. By contrast, diterpenoid and triterpenoid attenuated pulmonary edema and ameliorated ALI in LPS-induced ALI rats compared with the control vehicle. We presumed that the differences among the groups might be induced by the sample size gap and underlying publication bias. Of course, more studies are needed to confirm this speculation. Moreover, a low dose (≤10 µmol/kg) of diterpenoid and triterpenoid via intraperitoneal injection showed a significant outcome for decreasing lung wet-to-dry weight ratio. To our knowledge, this meta-analysis is an initial assessment of the therapeutic efficiency of terpenoids on LPS-induced ALI in rats. The total outcomes might offer a valuable reference to the future preventive and therapeutic use of terpenoids in human ALI.

In the overall analysis, there was no heterogeneity among studies. However, among studies in the subgroups of diterpenoid and low-dose terpenoid, it had a low degree of heterogeneity. Possible origin of heterogeneity contained strain of rat, route of LPS, and interval between LPS administration and sacrifice, each of which can influence the progression of ALI (Chen et al., 2010).

The meta-analysis is coupled with some restrictions. First, the correlation between the outcomes and humans is restrained by the differences between species in the development of pulmonary edema. The LPS-induced ALI animal model is well reputable, and the main characteristics and development of pulmonary edema in humans and rats emerge similar, but to a certain extent, it is different in pathogenesis (Tomashefski, 2000). We realize that an LPS-induced ALI animal model duplicates a lot of pathological features, but it is still relatively simple and rapid to reproduce the pathologic processes of humans (Matute-Bello et al., 2008). Second, other animal models of ALI were not included in this meta-analysis, such as pigs and sheep, which are easier to be induced by LPS because they have a more sensitive pulmonary hypertensive response and a higher speed circulation than rats (Warner et al., 1988; Schmidhammer et al., 2006). Third, only the lung wet-to-dry weight ratio was extracted to investigate the effects of terpenoids on ALI. Other parameters, such as proinflammatory cytokines, including tumor necrosis factor and interleukin, lung injury score, and lung-to-body weight ratio, were not considered (Eastwood et al., 2015; Butt et al., 2016). Fourth, hemiterpenoids, sesterterpenoids, and other terpenoids were not investigated in the analysis. In addition, more studies based on a huge number of samples and large-scale animal models are still necessary for determining whether terpenoids are effective for attenuating ALI in humans.

This meta-analysis showed that terpenoid is beneficial for alleviating ALI in rats. In particular, low-dose (≤10 µmol/kg) diterpenoid and triterpenoid significantly decrease lung wet-to-dry weight ratio in rats via intraperitoneal injection. We need more well-designed, prospective, and extensive animal model research to expand our understanding of the mechanisms of terpenoids in ALI treatment. We still need randomized controlled trials in humans to demonstrate the clinical benefit of terpenoids in the prevention and therapy of ALI.

## Data Availability

The original contributions presented in the study are included in the article/supplementary material; further inquiries can be directed to the corresponding authors.
